# The effectiveness of salicylic acid plasters compared with ‘usual’ scalpel debridement of corns: a randomised controlled trial

**DOI:** 10.1186/1757-1146-6-40

**Published:** 2013-09-24

**Authors:** Lisa J Farndon, Wesley Vernon, Stephen J Walters, Simon Dixon, Mike Bradburn, Michael Concannon, Julia Potter

**Affiliations:** 1 Podiatry Services, Jordanthorpe Health Centre, 1 Dyche Close, Sheffield Teaching Hospitals NHS Foundation Trust, Sheffield S8 8DJ, England; 2 School of Health and Related Research (ScHARR), The University of Sheffield, Regent Court, 30 Regent Street, Sheffield S1 4DA, England; 3 School of Human and Health Sciences, The University of Huddersfield, Queensgate, Huddersfield HD1 3DH, England; 4 School of Health Professions and Rehabilitation Sciences, University of Southampton, Highfield, Southampton SO17 1BJ, England

**Keywords:** Foot, Pain, Podiatry, Callosities, Debridement

## Abstract

**Background:**

Corns are a common foot problem and surveys have indicated that between 14-48% of people suffer from them. Many of these will seek podiatry treatment, however there is little evidence to indicate which current treatments provide long term resolution. This study compared ‘usual’ treatment (enucleation with a scalpel) with the application of 40% salicylic acid plasters to corns to investigate which is the most effective in terms of clinical, economic and patient-centred outcomes.

**Methods:**

A parallel-group randomised controlled trial was carried out in two centres where adults who presented with one or more corns and who met the inclusion criteria were allocated to either ‘usual’ scalpel debridement or corn plaster treatment. All participants had measurements of corn size, pain using a 100 mm visual analogue scale (VAS) and health-related quality of life (EQ-5D) measures by an independent podiatrist, blind to treatment allocation at baseline, 3, 6, 9 and 12 months.

**Results:**

202 participants were randomised to receive scalpel debridement or corn plaster treatment (101 in each group). At 3 months 34% (32/95) of corns had completely resolved in the corn plaster group compared with 21% (20/94) in the scalpel group (*p* = 0.044), and 83% (79/95) had reduced in size in the corn plaster group compared with 56% (53/94) in the scalpel group (*p* < 0.001). At 12 months, time to corn recurrence was longer in the corn plaster group (*p* < 0.001). Pain from the corns was significantly lower in the corn plaster group at 3 months (*p* < 0.001) and EQ-5D scores changed (improved), from baseline, by 0.09 (SD ±0.31) and 0.01 (SD ±0.25) points in the corn plaster and scalpel groups respectively (*p* = 0.056). By month 12, EQ-5D scores had changed by 0.12 and −0.05 in the corn plaster and scalpel groups respectively (*p* = 0.005). The EQ-5D, VAS scores and the four domains of the Foot Disability Scale were similar in both groups at 3 and 12 months. The economic analysis indicated that corn plasters were a cost effective intervention.

**Conclusions:**

The use of corn plasters was associated with a higher proportion of resolved corns, a prolonged time to corn recurrence, less pain and reduced corn size over the first 6 months in comparison with ‘usual’ scalpel treatment and this intervention was cost effective. Used under supervision of a podiatrist on appropriate patients, corn plasters offer an effective alternative to scalpel debridement.

## Background

Approximately 3.6% of the UK population may be seen by NHS podiatrists [1] and corns account for a large part of podiatrists’ everyday workload [2]. The incidence of corns has been reported at between 14-48% [3] and pain associated with foot problems including corns has been cited as the main reason for people to seek podiatry treatment [4-6]. Corns can also reduce mobility and health-related quality of life for some individuals [7].

Despite corns being such a common foot complaint, there is little evidence for the most effective treatment for long term resolution. The ‘usual’ treatment is removal with a scalpel by a podiatrist, and this is often repeated at regular intervals as the corn re-occurs.

There is evidence to suggest that plasters containing salicylic acid for the treatment of corns have been available for nearly 200 years [8] and continue to be used within the general population as a self treatment. Studies have found that corn plasters can be more effective than a placebo and result in between 62-95% removal of corns [9,10]. The concentration of salicylic acid within the plasters ranges from 10-50%, with the optimum formulation recommended at 40% [11]. The time the corn plaster needs to be adhered to the corn has also been investigated and a seven day application has been shown to produce the highest number of corn removals [10]. Podiatrists however, are still reluctant to use them or recommend them as a self treatment, due to a belief of the potential for complications [12].

Although there is evidence that corn plasters resolve corns, this is the first large scale study to investigate their effectiveness. No comparisons have been made between corn plasters and the ‘usual’ scalpel removal of corns, in terms of patient benefit or cost effectiveness, and the majority of evidence has only considered immediate and short-term outcomes. This study compared the clinical and cost effectiveness of 40% salicylic acid corn plasters with scalpel debridement for corns for up to 12 months.

## Methods

This was a multi-centre parallel-group randomised controlled trial carried out in one podiatry service and one school of podiatry in the United Kingdom. The study obtained ethical approval from Leicestershire, Northamptonshire and Rutland Research Ethics Committee 2 (09/H0402/07), Medicines and Healthcare Products Regulatory Agency (MHRA) and local research governance approval. The study sponsor was Sheffield Health and Social Research Consortium. All participants gave informed consent before taking part in the trial.

### Participants and randomisation

Adults with one or more corns and able to give informed consent, were recruited to the study from existing podiatry patients in one National Health Service (NHS) podiatry service. Recruitment was later expanded to include patients attending one university school of podiatry clinic and the local population of the two localities to increase the number of eligible participants. The recruitment period was also extended for the same reason and was between September 2009 and October 2011. Participants were excluded if they had diabetes or rheumatoid arthritis; poor peripheral circulation or peripheral neuropathy; a history of foot ulceration; were taking oral steroid medication; had a marked dermatological condition (e.g. eczema, psoriasis, etc.), were allergic to zinc oxide plaster, salicylic acid, peanuts or soya; were unable to reach their own feet; had callus rather than corns, or corns that were infected or neurovascular; or were pregnant or breastfeeding.

Treatment allocations were concealed by a secure web-based randomisation system hosted within the Sheffield Clinical Trials Research Unit. Eligible patients who gave written consent were allocated treatment by a simple randomisation sequence.

### Intervention

#### Corn plaster

Participants randomised to the corn plaster group had a corn plaster (Carnation corn plasters®, Cuxson Gerrard & Co) applied to each corn (up to three corns per person) by the podiatrist and appropriate dressings applied if necessary to keep the plasters in place. The plasters contain 40% salicylic acid. Participants with multiple corns were asked to nominate one corn post-randomisation, usually the most painful or largest, which was classed as the ‘index’ corn and used as the primary outcome measure. Each participant was advised to keep the corn plasters dry until they returned a week later. Advice was given about removal and neutralisation of the salicylic acid with a warm salt foot bath if any adverse reaction was experienced. At the next appointment new corn plasters were applied if the corns were still present and this was repeated for up to four weeks or until the corns could be lifted off via blunt debridement by the podiatrist. The participants then returned at intervals of three months for a year and received ‘usual’ scalpel debridement if any of the corns remained. Any participant that had a reaction to the corn plaster or was unable to tolerate the treatment had the corn plasters removed and resorted to the ‘usual’ scalpel treatment group.

#### ‘Usual’ scalpel debridement

Participants randomised to the ‘usual’ scalpel debridement group were asked to identify an ‘index’ corn post-randomisation if they had more than one corn and this was used as the primary outcome measure. All corns were treated with a scalpel by a podiatrist and this was repeated at three monthly intervals for twelve months if the corns were still present.

Standard foot health and footwear education was given at each appointment to all participants.

#### Outcomes

The primary outcome was the presence at three months post-randomisation of an improved index corn. For the purpose of the analyses, two definitions of an improved corn were used: *resolution* (the index corn had not recurred or was measured at a width of less than 1 mm) and *reduction* (the index corn measuring a width less than the baseline measurement). Both were assessed by an independent podiatrist ‘blind’ to the treatment group who measured the site and size of the corns at baseline and at each three monthly follow-up appointments. Secondary outcomes were pain experienced from corns; health-related quality of life and foot related disability, patient and podiatrist satisfaction with the treatments, and cost effectiveness. Participants were asked to complete a pain visual analogue scale (100 mm VAS) for the index corn, the EQ-5D health-related quality of life questionnaire [13] and the Foot Disability Questionnaire [14] at baseline and at three monthly intervals (3, 6, 9 and 12 months). The EQ-5D is a measure of health utility derived from the answer to five questions, in which 1 relates to perfect health and 0 to death; negative values (i.e. “a state worse than death”) are allowed, with the minimum possible score being −0.59. The EQ-5D VAS is a visual analogue scale measure of current state of health (0 = worst imaginable, 100 = best imaginable). A simple satisfaction questionnaire was also completed at each post-baseline follow up for patients and one questionnaire was given to podiatrists delivering the interventions at the end of the study. The treating podiatrist recorded the time taken for each appointment and the type and number of podiatry instruments or corn plasters used for each treatment on a resource form.

#### Sample size

The primary outcome was the presence, at 3 months post randomisation, of an unhealed or recurrent corn that required further or on-going treatment. Two studies have shown that between 60-90% of corns can be resolved at 3 months using corn plasters [9,10]. We assumed that there could be a 3 month recurrence rate of up to 40% in the corn plaster group and 60% in the scalpel group and that a 20% absolute difference in corn recurrence rates between the groups is of clinical and practical importance, which corresponds to an odds ratio (OR) of 2.25. To have 80% power to detect this difference in corn recurrence healing/rates between the two groups as statistically significant at the 5% (two-sided level), required 100 participants in each treatment arm.

### Statistical analysis

The difference in the proportion of participants with a healed index corn at each time-point was analysed using logistic regression, adjusting for centre and size of the index corn at baseline. The sensitivity of findings to missing data was assessed by several imputation strategies including best case/worst case scenario and multiple imputation as described in the results. Time to corn recurrence, defined as the time at which the corn size was greater than or equal to the size at baseline, was analysed using Cox regression with the same covariates. The corn size, pain and health-related quality of life outcomes at each time-point were analysed using the same covariates. Statistical significance was set at *p* < 0.05.

A cost-utility analysis was undertaken to assess the cost-effectiveness of corn plasters compared to ‘usual’ scalpel debridement considering the economic dimensions from the perspective of the podiatry service. The timeframe of the primary analysis is the same as the clinical analysis (12 months). Cost and outcome data were collected for individual participants in the trial. Resource use covers number and length of podiatry appointments and equipment used. Unit costs were identified for one minute of a podiatrist’s time and associated consumables. Podiatrist time was costed using Curtis [15] and inflated to 2010/11 prices, whilst consumable prices were obtained from Sheffield Teaching Hospitals (NHS) Foundation Trust. Quality-adjusted life years (QALYs) were calculated from the EQ-5D scores using the trapezium rule, with QALYs gained calculated by subtracting baseline utility.

The baseline economic analysis was based on costs and outcomes observed in the trial. However, this is problematic as those visits occurring after resolution of the corn would not take place in practice; these were therefore excluded in a subsequent analysis of ‘normal practice’. There were further problems due to missing data. In an attempt to overcome this, a further analysis of normal practice was undertaken; with missing data imputed using the last-observation carried forward method.

Patient and podiatrist satisfaction were reported as frequency of response categories. Patient satisfaction was compared between treatments using the chi-squared test.

## Results

**Figure 1:**
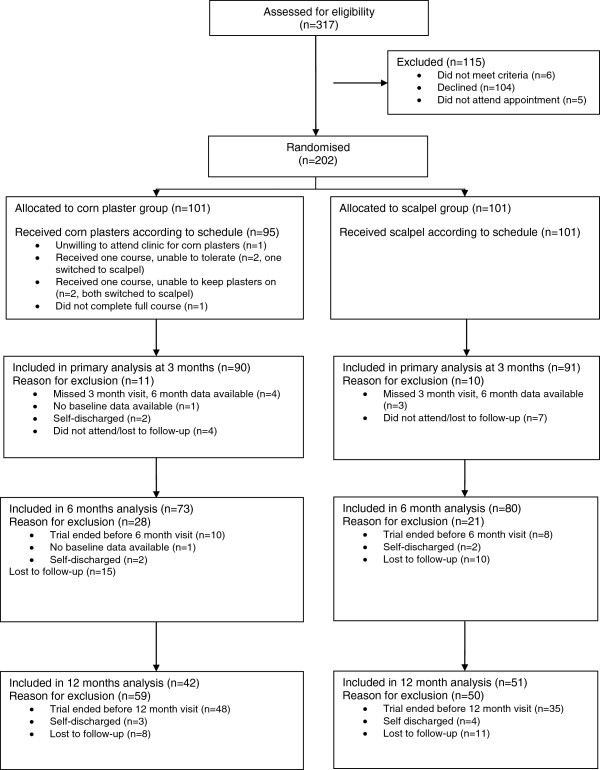
Flow of participants through the trial.

In total, 202 participants were recruited and randomised (101 per group). Figure 1 shows the flow of participants through the trial. Twenty participants (10%) had no corn assessment at 3 months; of these, 7 attended at 6 months and 13 (6%) were lost to follow-up completely. The primary analyses took a conservative approach to the former: index corns that had improved at 6 months were assumed also to have improved at 3 months and otherwise not. The remaining 13 participants that were lost to follow-up were not included in the primary analysis (i.e. not intention-to-treat analysis). Further analyses assessed the sensitivity of the findings to other imputation methods and assumptions. The baseline demographic and clinical characteristics of the trial participants were broadly similar in the corn plaster and scalpel debridement groups (Table 1).

**Table 1:** Baseline characteristics of participants

		**Corn Plaster**		**Scalpel**		**Total**
		**(n = 101)**		**(n = 101)**		**(N = 202)**
Gender						
*Male*		42 (42%)		42 (42%)		84 (42%)
*Female*		59 (58%)		59 (58%)		118 (58%)
Centre						
*1*		58 (58%)		53 (53%)		111 (54%)
*2*		1 (1%)		1 (1%)		2 (1%)
*3*		7 (7%)		4 (4%)		11 (5%)
*4*		5 (5%)		4 (4%)		9 (4%)
*5*		10 (10%)		13 (14%)		23 (12%)
*6*		7 (7%)		6 (6%)		13 (6%)
*7*		13 (13%)		20 (20%)		33 (16%)
Number of corns evaluated						
*1*		48 (48%)		66 (65%)		114 (56%)
*2*		28 (28%)		23 (23%)		51 (25%)
*3*		24 (24%)		12 (12%)		36 (18%)
*Missing*		1 (1%)		0		1 (<1%)
	**n**	**Mean (SD)**	**n**	**Mean (SD)**	**N**	**Mean (SD)**
Age	101	58.5 (15.6)	101	59.7 (17.5)	202	59.1 (16.6)
Size of index corn (mm)	99	3.9 (1.7)	101	3.8 (1.8)	200	3.8 (1.8)
VAS pain (0–10)	100	5.71 (2.92)	101	4.87 (2.97)	201	5.29 (2.97)
EQ5D tariff	98	0.64 (0.30)	101	0.68 (0.28)	199	0.66 (0.29)
EQ5D VAS (0–100)	100	74.3 (19.6)	99	73.5 (20.3)	199	73.9 (19.9)

### Primary outcome

At 3 months post-randomisation, 34% (32/95) of participants had a resolved corn in the corn plaster group compared with 21% (20/94) in the scalpel group (OR 2.00, 95% CI: 1.02 to 3.93, *p* = 0.044) in favour of the corn plaster group, indicating that the odds of a completely healed index corn among participants allocated corn plasters was twice that of that of participants in the scalpel group at three months post-randomisation. Eighty-three per cent (79/95) of participants in the corn plaster group and 56% (53/94) in the scalpel group had a reduction in corn size at three months (OR = 4.42, 95% CI 2.17 to 8.97; *p* < 0.001) (see Table 2).

**Table 2:** Comparison of corns that were healed and improved over 12 months by group

	**Corn Plaster**	**Scalpel**	**Difference**	
	**(n = 101)**	**(n = 101)**		
No (%) with completely healed index corn*			OR (95% CI)***	**P**
3 months	32/95 (34%)	20/94 (21%)	2.00 (1.02,3.93)	0.044
6 months	27/74 (36%)	24/80 (30%)	1.59 (0.76,3.32)	0.215
9 months	18/55 (33%)	22/64 (34%)	0.99 (0.44,2.20)	0.973
12 months	20/43 (47%)	16/51 (31%)	1.94 (0.78,4.79)	0.153
No (%) with improved index corn**			OR (95% CI)***	**P**
3 months	79/95 (83%)	53/94 (56%)	4.42 (2.17,8.97)	<0.001
6 months	62/74 (84%)	54/80 (68%)	2.74 (1.19,6.28)	0.017
9 months	44/55 (80%)	49/64 (77%)	1.24 (0.48,3.25)	0.655
12 months	33/43 (77%)	39/51 (76%)	0.97 (0.34,2.75)	0.948
**Mean (SD) size of index corn (mm)**			Mean diff. (95% CI)****	**P**
Baseline	3.9 (1.7)	3.8 (1.8)		
3 months	1.7 (1.6)	2.7 (2.3)	−1.0 (−1.5, 0.5)	<0.001
6 months	1.7 (1.6)	2.4 (2.2)	−0.7 (−1.2, -0.2)	0.006
9 months	1.7 (1.6)	2.2 (2.1)	−0.5 (−1.2, 0.1)	0.107
12 months	1.3 (1.5)	2.3 (2.2)	−1.0 (−1.7, -0.2)	0.010

*Defined as a corn size <1 mm. ** Defined as a reduction from baseline in corn size.

***From logistic regression, adjusted for centre and baseline corn size.

****From analysis of covariance on change from baseline, adjusted for centre and baseline corn size.

The difference in complete corn resolution was not robust to conservative imputation strategies. Of the methods used, only multiple imputation including treatment group as a predictor approached a statistically significant effect (OR = 1.97, 95% CI 1.00 to 3.89, *p* = 0.050). By contrast, the statistical significance of the corn size reduction was maintained even in the most extreme missing data imputation (index corns reduced in the scalpel group, not reduced in the corn plaster group).

### Secondary outcomes

**Figure 2:**
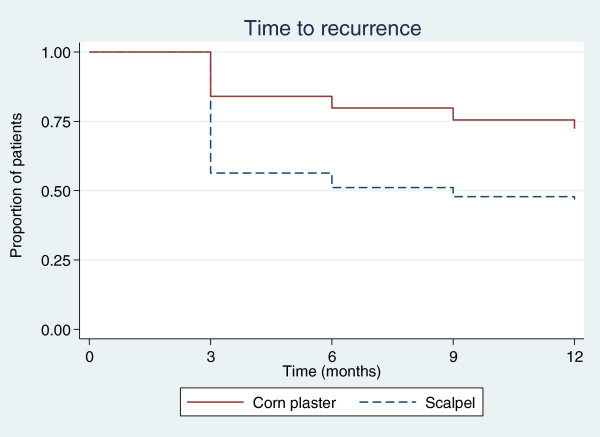
Time to corn recurrence.

**Figure 3:**
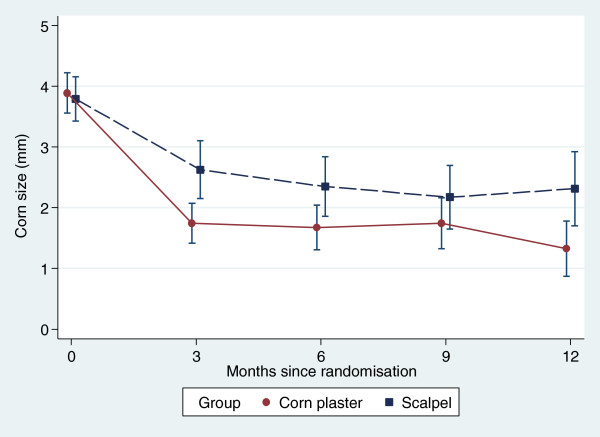
Corn size at different time points (means and standards errors).

Recurrent corns were retreated by scalpel debridement where needed, and consequently the differences between the two groups diminished with time. At 6 months, the percentage of participants with a completely resolved corn was 36% in the corn plaster group and 30% in the scalpel group (adjusted OR = 1.59, 95% CI 0.76, to 3.32, *p* = 0.215). The corresponding percentages for size reduction were 84% versus 68% (adjusted OR = 2.74, 95% CI 1.19 to 6.28, *p* = 0.017). The size of the index corn was reduced over the duration of the trial, and the time taken for the corn to be considered recurrent (i.e. to revert to its size at baseline) was longer (hazard ratio = 0.40, 95% CI 0.24 to 0.65, *p* < 0.001; Figures 2 and 3). Pain measured by the 100 mm VAS was significantly lower in the corn plaster group at 3 months (mean difference = 1.0; 95% CI −1.5 to −0.6, *p* < 0.001).

At 3 months, mean EQ-5D scores had improved by 0.09 (SD ±0.31) and 0.01 (SD ±0.25) from baseline in the corn plaster and scalpel groups respectively, equating to a mean difference between the groups of 0.08 (95% CI: 0.00 to 0.16, p = 0.056). At 12 months, the EQ-5D scores had changed by 0.12 and −0.05 in the corn plaster and scalpel groups respectively (mean difference = 0.17, 95% CI 0.05 to 0.29, p = 0.005). The EQ-5D VAS and the four domains of the Foot Disability scale were similar across the groups over the course of the study. Mean EQ-5D scores had improved from baseline by 0.09 (SD ±0.31) and 0.01 (SD ±0.25) points in the corn plaster and scalpel groups respectively, equating to a mean difference between the groups of 0.08 (95% CI: 0.00 to 0.16, *p* = 0.056).

For the economic analysis, mean costs were UK£24 higher in the corn plaster group as these participants attended the clinic approximately 3 times more than those in the scalpel group. QALYs gained were slightly higher in the corn plaster group and the intervention was cost-effective at different funding thresholds. The typically used threshold of UK£20,000 for cost-effectiveness found that corn plasters were 98% cost effective [16].

Participant satisfaction was high for both treatment groups, with >85% stating they were either satisfied or very satisfied at each time point; no significant differences were observed at any time. Self-reported improvement was more common in the corn plaster group at 3 months (80% versus 56% in scalpel group, *p* = 0.001) but not thereafter; >50% of participants in both arms believed their corn had improved since the previous visit at month 6, 9 and 12. Nearly all participants (>95%) reported they would be happy to receive the same treatment again, irrespective of group. Of the eight research podiatrists that took part in this study, 7 out of 8 stated that they preferred corn plasters rather than scalpel treatment, the main reasons stated were ease of use and better outcomes for participants in terms of pain and healing of the corn.

### Compliance and adverse events

The majority of participants in the corn plaster group were able to tolerate the plasters and keep them in place as requested. There were 4 related adverse events in the corn plaster group, these were; maceration at the site, development of a haematoma, an aseptic breakdown and itching. They were rated as either mild or moderate and all resolved within a few days after the corn plasters were removed. No suspected unexpected serious adverse reactions were reported by participants in either group.

## Discussion

We compared the effectiveness of corn plasters with scalpel debridement for the treatment of corns and found that corn plasters were associated with a higher proportion of healed corns, a prolonged time to recurrence, less pain and reduced corn size over the first 6 months compared with standard care (scalpel treatment). Long term benefits indicated that those who had corn plasters were 60% less likely to have recurrence of a corn at 12 months. There was some evidence of an increase in QALYs, and that the intervention is likely to be cost effective.

There were a number of differences in this trial compared to previous studies. This study included corns on all sites of the foot (dorsal and plantar aspects) and used ‘usual’ scalpel treatment as the comparator, whereas prior research has only included digital corns and either no comparator or a placebo [9-11,17]. This study is also the first to compare the long and short term effectiveness of corn plasters for up to a year. Lang et al’s study [10] was a randomised controlled trial, but participants applied their own corn plasters at daily intervals for up to 10 days and the mean size of corns at baseline was notably larger than in this trial (8.04 mm versus 3.90 mm). They also reported the number of enucleated (removed) corns using corn plasters at 10 days (62%), whereas our trial considered *effectiveness* to be a reduction in size or the continued resolution of corns at 3 months and up to 12 months. Potter’s study [11] was also a controlled trial and used a similar method, where podiatrists applied the corn plasters and carried out blunt debridement to remove the macerated lesions. In that study the plasters were reapplied 3 times per week for up to 2 weeks and the main outcome was corn resolution; a reduction in the size of the corn was not included.

Given that the corn plasters contain salicylic acid, we were particularly interested in any adverse effects. Our trial found that corn plasters were safe when applied under professional supervision and well tolerated by participants with only four related adverse events reported. Similar results relating to adverse events were found in Lang et al’s trial [10] and none were reported in Potter’s study [12]. Adverse events recorded were either mild or moderate and all resolved quickly.

The results of this trial need to be considered with the following limitations in mind. This trial did not investigate repeated corn plaster usage: participants randomised to corn plasters who subsequently required follow-up treatment received this with a scalpel. In addition, the recruitment was slower than expected, resulting in a curtailed follow-up of less than the planned 12 months for 83 (41%) of the participants and correspondingly an imprecise estimate of longer-term impact of corn plasters. On the positive side, the resultant missing data can be considered as random and therefore the figures presented are at least unbiased.

## Conclusion

The results of this trial show that corn plasters used under podiatry supervision are an effective treatment for corns in suitable participants with better outcomes, especially short term, when compared with scalpel debridement alone. Corn plasters were found to be safe and well tolerated by the majority of participants and most podiatrists thought that they were a more successful treatment than scalpel removal of corns. Though initially participants required more frequent visits, appointments were shorter, outcomes were improved, and there was an increased chance of resolution or reduction in corn size. This could lead to long-term benefits, not only in a better outcome for patients, but in terms of saved appointment times and discharge of patients. Cost analysis also showed that this treatment is cost effective.

## Competing interests

The authors declare that there were no competing interests.

## Authors’ contributions

WV, LF, and JP and CC designed the trial. WV and BK were joint Chief Investigators and LF was the trial manager. JM and CM designed the case report forms. TC designed the database and web based randomisation system in conjunction with EpiGenesys. AL was data manager. MB, SW and SD conducted the analysis. EL assisted with programming data checks and additional analyses. LF, WV, JP, MB, SW, MC and SD drafted and approved the final manuscript.
